# Progressive evolution of Streptococcus equi from Streptococcus equi subsp. zooepidemicus and adaption to equine hosts

**DOI:** 10.1099/mgen.0.001366

**Published:** 2025-03-28

**Authors:** Hayley J. Wilson, Jianbao Dong, Andries J. van Tonder, Christopher Ruis, Noémie Lefrancq, Abigail McGlennon, Carla Bustos, Sara Frosth, Albertine Léon, Adam M. Blanchard, Matthew Holden, Andrew S. Waller, Julian Parkhill

**Affiliations:** 1 PHG Foundation, linked exempt charity of University of Cambridge, Cambridge, UK; 2 Department of Veterinary Medicine, University of Cambridge, Cambridge, UK; 3 College of Animal Science and Technology, Qingdao Agricultural University, Qingdao, PR China; 4 Victor Phillip Dahdaleh Heart & Lung Research Institute, University of Cambridge, Cambridge, UK; 5 Department of Genetics, University of Cambridge, Cambridge, UK; 6 Royal Veterinary College, Hatfield, Hertfordshire AL9 7TA, UK; 7 EIDS, Department of Veterinary Medicine, University of Cambridge, Cambridge, UK; 8 Facultad de Ciencias Veterinarias, Cátedra de Enfermedades Infecciosas, Universidad de Buenos Aires, Buenos Aires, Argentina; 9 Consejo Nacional de Investigaciones Científicas y Técnicas (CONICET), Buenos Aires, Argentina; 10 Department of Animal Biosciences, Swedish University of Agricultural Sciences, P.O. Box 7023, 750 07 Uppsala, Sweden; 11 LABÉO, Research Department, St Contest, Caen, France; 12 Normandie Univ, UNICAEN, INSERM, DYNAMICURE UMR 1311, Caen, France; 13 School of Veterinary Medicine and Science, University of Nottingham, Sutton Bonington, UK; 14 Infection Group, School of Medicine, University of St Andrews, North Haugh, St Andrews, UK; 15 Intervacc AB, Stockholm, Sweden

**Keywords:** epidemiology, evolution, genomic, strangles, *Streptococcus equi*, zooepidemicus

## Abstract

*Streptococcus equi* subsp. *equi* causes the equine respiratory disease ‘strangles’, which is highly contagious, debilitating and costly to the equine industry. *S. equi* emerged from the ancestral *Streptococcus equi* subsp. *zooepidemicus* and continues to evolve and disseminate globally. Previous work has shown that there was a global population replacement around the beginning of the twentieth century, obscuring the early genetic events in this emergence. Here, we have used large-scale genomic analysis of *S. equi* and its ancestor *S. zooepidemicus* to identify evolutionary events, leading to the successful expansion of *S. equi*. One thousand two hundred one whole-genome sequences of *S. equi* were recovered from clinical samples or from data available in public databases. Seventy-four whole-genome sequences representative of the diversity of *S. zooepidemicus* were used to compare the gene content and examine the evolutionary emergence of *S. equi*. A dated Bayesian phylogeny was constructed, and ancestral state reconstruction was used to determine the order and timing of gene gain and loss events between the different species and between different *S. equi* lineages. Additionally, a newly developed framework was used to investigate the fitness of different *S. equi* lineages. We identified a novel *S. equi* lineage, comprising isolates from donkeys in Chinese farms, which diverged nearly 300 years ago, after the emergence of * S. equi* from *S. zooepidemicus*, but before the global sweep. Ancestral state reconstruction demonstrated that phage-encoded virulence factors *slaA*, *seeL* and *seeM* were acquired by the global *S. equi* after the divergence of the basal donkey lineage. We identified the equibactin locus in both *S. equi* populations, but not *S. zooepidemicus*, reinforcing its role as a key *S. equi* virulence mechanism involved in its initial emergence. Evidence of a further population sweep beginning in the early 2000s was detected in the UK. This clade now accounts for more than 80% of identified UK cases since 2016. Several sub-lineages demonstrated increased fitness, within which we identified the acquisition of a new, fifth prophage containing additional toxin genes. We definitively show that acquisition of the equibactin locus was a major determinant in *S. equi* becoming an equid-exclusive pathogen, but that other virulence factors were fixed by the population sweep at the beginning of the twentieth century. Evidence of a secondary population sweep in the UK and acquisition of further advantageous genes implies that *S. equi* is continuing to adapt, and therefore, continued investigations are required to determine further risks to the equine industry.

**Impact Statement**

Strangles, caused by *Streptococcus equi* subsp. *equi*, is highly contagious and results in high morbidity and economic losses to the equine industry. Previous genomic analyses determined that *S. equi* evolved into a successful equine host-restricted pathogen due to the acquisition of several prophages and integrative conjugative elements. It also identified a potential genetic population sweep in the early twentieth century. We have combined multiple published genomic datasets and newly sequenced isolates to create the largest genomic dataset of *S. equi* to date. This has enabled us to shed new light on the evolutionary events that led to the successful pathogen we see infecting equines currently. Our work provides a deeper understanding of these processes and provides groundwork for further genomic analyses of contemporary *S. equi*. We have demonstrated that * S. equi* is continuing to evolve by identifying the potential acquisition of another prophage in a proportion of isolates and a second ongoing genetic population sweep.

## Data Summary

All genomic sequence data have been deposited in the European Nucleotide Archive under the respective project identifiers. Sequences new to this project can be found under project PRJEB77744 with accession numbers for each isolate detailed in Data S1 (available in the online Supplementary Material). The data can be accessed via this link https://www.ebi.ac.uk/ena/browser/view/PRJEB77744. All previously published data can be found publicly using the accession numbers detailed in Data S1. All supporting data, code and protocols have been provided within the article or through supplementary data files. One supplementary figure, one supplementary methods document and one supplementary data table are available with the online version of this article.

## Introduction

Strangles in horses was first identified as being caused by *Streptococcus equi* subsp. *equi* (*S. equi*) in 1888, but reports of the disease date to the thirteenth century [1,2]. *S. equi* is highly contagious and capable of quickly infecting a whole herd, with up to a 10% mortality rate, resulting in a considerable economic loss [3]. Rupture of the disease-typical abscesses releases infective material into the environment promoting rapid onward transmission to in-contact animals. Incomplete drainage of abscesses in the retropharyngeal lymph nodes, which rupture into the guttural pouch, can lead to the formation of chondroids and a persistent subclinical ‘carrier’ state that facilitates transmission of *S. equi* to new populations of horses [4].

Despite the implication from the species and subspecies nomenclature, molecular evidence has shown that *S. equi* is a clonal lineage, which evolved from the more diverse ancestral species *Streptococcus equi*. subsp. *zooepidemicus* [5,6]. * S. zooepidemicus* is an opportunistic pathogen of mammals causing a broad range of diseases including respiratory infections, keratitis, pneumonia, abortion, renal disease and septicaemia [7]. The loss of genetic material during the evolution of *S. equi* from *S. zooepidemicus* is believed to have led to its host restriction, whilst the gain of mobile genetic elements is proposed to have transformed the ability of this obligate pathogen to cause disease [6].

The genomes of *S. equi* isolates typically contain four prophages that are usually absent from the genomes of *S. zooepidemicus* but are similar to counterparts encoded by the host-restricted human pathogen *Streptococcus pyogenes*, indicating a common phage pool [6]. The prophages carry virulence determinants including phospholipase A_2_ toxin, SlaA [8], SeeL (SpeK), SeeM (SpeL), SeeH (SpeH) and SeeI (SpeI) [6]. Furthermore, the genome of *S. equi* encodes an integrative conjugative element, ICE*Se2*, comprising a large gene locus responsible for the production and secretion of the putative siderophore equibactin [9]. The production of equibactin enhances the ability of *S. equi* to acquire iron, significantly increasing the virulence of this pathogen in horses [9,10]. However, deletions of all, or part, of the equibactin locus were observed in isolates recovered from some persistently infected carrier horses, suggesting that the locus is important for acute infection but may be dispensable/disadvantageous for longer-term, subclinical carriage [10].

Genomic analyses have demonstrated that *S. equi* exhibits a low level of diversity relative to its global distribution [10,12], which is problematic when it comes to partitioning the population for analysis and epidemiological investigations. Multilocus sequence typing (MLST) provides poor resolution for *S. equi*. Sequence types (STs) are determined using the *S. zooepidemicus* MLST scheme, with fewer than ten STs, all of which are single, or double, locus variants of ST179 [5]. Globally, ST179 and the more recently identified ST151 account for more than 95% of isolates identified in multiple collections. Genome-based phylogenetic comparisons also support the observation of restricted diversity [10,11, 13].

More recently, the use of Bayesian Analysis of Population Structures (BAPS) to infer genetic cluster identified six BAPS clusters, which demonstrated the geographical distribution of lineages and transmission on a global scale [11]. Despite this widespread transmission, the mean core genome SNP difference between clusters was less than 100, which was attributed to a large-scale population replacement or genetic bottleneck estimated to have occurred during the early twentieth century [10]. However, the genetic events that drove this population replacement are unknown, as is the population structure and genetic composition of *S. equi* before the replacement.

Since 2018, a Horse Trust funded initiative, Surveillance of Equine Strangles (SES) has been building a comprehensive strangles surveillance network in the UK [14]. Isolates of *S. equi* and their linked epidemiological data have been collated to provide increased clarity on circulating strains. Strangles illness in donkeys is infrequent compared to horses; however, donkeys that become exposed to *S. equi* through contact with horses can also be affected. Previously undescribed outbreaks of severe disease, leading to the death of 12.5% of young donkeys in affected Chinese farms, have been recently reported, from which a novel SeM-type of *S. equi* was isolated [15]. Additionally, a comparison of the genomes of three donkey isolates to the *Se*4047 genome was reported in 2023 [16].

Here, we aimed to create the largest genomic dataset of *S. equi* to date and utilize the information contained within it to shed unprecedented light on the evolution of *S. equi*. By combining data from both horses and donkeys, we have identified key gene acquisition events that led to the successful evolution of *S. equi* from *S. zooepidemicus* and resolved those occurring during the initial emergence of *S. equi*, from those associated with the genetic bottleneck in the early twentieth century. We have also identified evidence of further genetic events that potentially are leading to another population sweep in *S. equi* of a lineage with increased fitness that began in the UK and has begun to spread globally.

## Methods

### Bacterial isolates

After the removal of isolates that did not meet species or quality criteria (see below), the genomes of 1201 isolates were combined from multiple sources to create the study dataset. The 639 isolates from the dataset collated by Mitchell *et al*. [11] were included in this collection. The remaining 562 isolates were sourced from collaborators and publicly available data (Supplementary Methods). Closed genomes sequenced on an Oxford Nanopore Technologies Minion sequencer were provided by Associate Professor Adam Blanchard from the University of Nottingham. The full details of each isolate used can be found in Data S1.

### Sequence read quality control

Sequencing reads for each isolate were examined for quality metrics using BacQC v1.0 (https://github.com/avantonder/bacQC). Full pipeline details can be found in the Supplementary Methods. Fourteen *S*. *zooepidemicus* isolates were removed due to mistakenly being identified as *S. equi*. One isolate was removed due to being *Streptococcus dysgalactiae*. Thirteen genomes were excluded due to poor-quality sequencing.

Sequence reads were mapped to the *Se*4047 reference genome (accession number: FM204883) and assembled using Snippy v3.1 (https://github.com/tseemann/snippy). Output files produced by Snippy did not retain missing base calls (i.e. Ns), which were written to a separate file. Therefore, to obtain complete and accurately mapped assemblies of each isolate, a custom python script was used to combine the two data files and create each individual alignment (https://github.com/francesccoll/scripts/blob/main/create_snippy_consensus.py). The resulting output files were then concatenated to create a multi-fasta mapped alignment of the 1201 isolates for further downstream use.

### fastbaps clustering

We inferred genetic clusters for the population using BAPS and the fastbaps v1.0.8 algorithm [17], which requires a phylogeny reconstructed using polymorphic sites as input. A core genome alignment was created by removing the prophages and integrative conjugative elements (ICE) using a Sanger-Pathogens script (https://github.com/sanger-pathogens/remove_blocks_from_aln) and Gubbins v3.1.4 [18] removed regions of recombination. The ‘filtered_polymorphic_sites.fasta’ output alignment produced by Gubbins was then used to reconstruct the phylogeny using IQ-TREE v2.2.0 [19,20] with ultrafast bootstrapping [21] (-bb 1000), details of the constant sites (-fconst) and a general time reversible model. The resultant alignment and phylogenetic tree were used to run the fastbaps R package [17]. The ‘baps’ prior was used, and data were constrained within the submitted phylogeny.

### Genomic comparisons between *S. zooepidemicus* and *S. equi*

To investigate gene gain and loss during the evolution of *S. equi* from *S. zooepidemicus*, we utilized a dataset of 804 global * S. zooepidemicus* sequenced and assembled for the former Animal Health Trust. Following the preparation of a core gene alignment (see Supplementary Methods), custom python scripts were then used to perform maximum parsimony ancestral state reconstruction (https://github.com/pathgenevocam/panaroo_team), which produced information on the gene gain and loss events and inferred plausible ancestral states across the phylogeny. To filter the results to a manageable size, a node-labelled phylogenetic tree was used to identify key branches and events within the phylogeny. Genes identified as key gain or loss events were checked for accuracy using Cytoscape [22] v3.8.2 and blast (https://blast.ncbi.nlm.nih.gov/Blast.cgi).

### Dating the phylogeny

BEAST2 v2.6.6 [23] was used for Bayesian reconstruction of the phylogeny to identify the time to most recent common ancestor (TMRCA) for the *S. equi* dataset. Full details of the data preparation and parameters used can be found in the Supplementary Methods. The data were run in triplicate until effective sample size values for all parameters exceeded 200. A date-tip permutation test was performed on ten replicates with randomized date-tip combinations to confirm an accurate signal from the BEAST2 analysis. The BEAST2 [23] package ‘logcombiner’ was used with a 10% burn-in to combine the log file and tree file replicates. TreeAnnotator [23] was then used to create a maximum clade credibility (MCC) tree from these data.

### Fitness index

To analyse the fitness dynamics of the *S. equi* sequence data, we used only isolates collected from the UK due to a lack of global isolates since 2020. A time-resolved phylogeny was created as seen above in ‘Dating the phylogeny’ using 587 isolates collected from the UK between 1990 and 2022. We then utilized a newly developed analytical framework (full methodology can be found in Lefrancq *et al.* [24]) that summarizes changes in the composition of populations in phylogenetic trees at every time point (see Supplementary Methods).

### Ancestral state reconstruction in *S. equi*

To investigate potential drivers of the successful expansion of FB7/ST151 in the UK, we again utilized ancestral state reconstruction. Panaroo v1.3.2 [25] was used to create a core gene alignment of the annotation data for each *S. equi* isolate. SNPs in this alignment were identified, and a phylogenetic tree was created on the SNP alignment using IQ-TREE [19,26] with the same settings used for the combined *S. equi* and *S. zooepidemicus* data. The data were then used as input for the custom python scripts used previously with the combined *S. equi* and *S. zooepidemicus* data (https://github.com/pathgenevocam/panaroo_team). As before, key nodes within the phylogenetic tree were identified, and the gene gain and loss events associated with them were investigated.

### Prevalence of mobile genetic elements in the dataset

To determine the stability or presence and absence of the mobile genetic elements (MGEs) within the population, we used Blat v36 [27] to screen the isolates for the presence of specific markers for each prophage and ICE. To prevent excessive cross-matching and to circumvent issues with accurately assembling prophages from short-read data, we used proxy markers for the presence of each element. ΦSeq1 and ICE*Se1* are not known to carry any virulence cargo genes; therefore, we did not include them in our searches. *slaA* was used to search for the presence of ΦSeq2. The superantigen genes *seeL* and *seeM* were used to search for ΦSeq3 and *seeH* and *seeI* were used for ΦSeq4. The equibactin locus genes *eqbA* to *eqbN* were used to identify the presence of ICE*Se2.* Each sequence was obtained from the original annotation of the reference strain *Se*4047. The *seeL* variant was examined at the nt level using Seaview [28].

### Data visualization

R v4.0.4, ggplot v3.5.1 [29,30], ggtree v3.4.4 [31] and gggenes v0.5.1 (https://cran.r-project.org/web/packages/gggenes/gggenes.pdf) were used to create the figures in this manuscript.

## Results

### Partitioning/clustering the population

We initially assessed multilocus STs in the complete genome collection of 1201 isolates and found 97.6% belonged to just two STs, ST179 and ST151, highlighting the poor resolution of this method for *S. equi.* Analysis using fastbaps resulted in ten clusters (FB1–10). BAPS has previously been used to sub-divide populations of *S. equi*, although the groups produced previously do not correlate exactly with the newer groups created in our expanded dataset (Data S1).

**Fig. 1.**
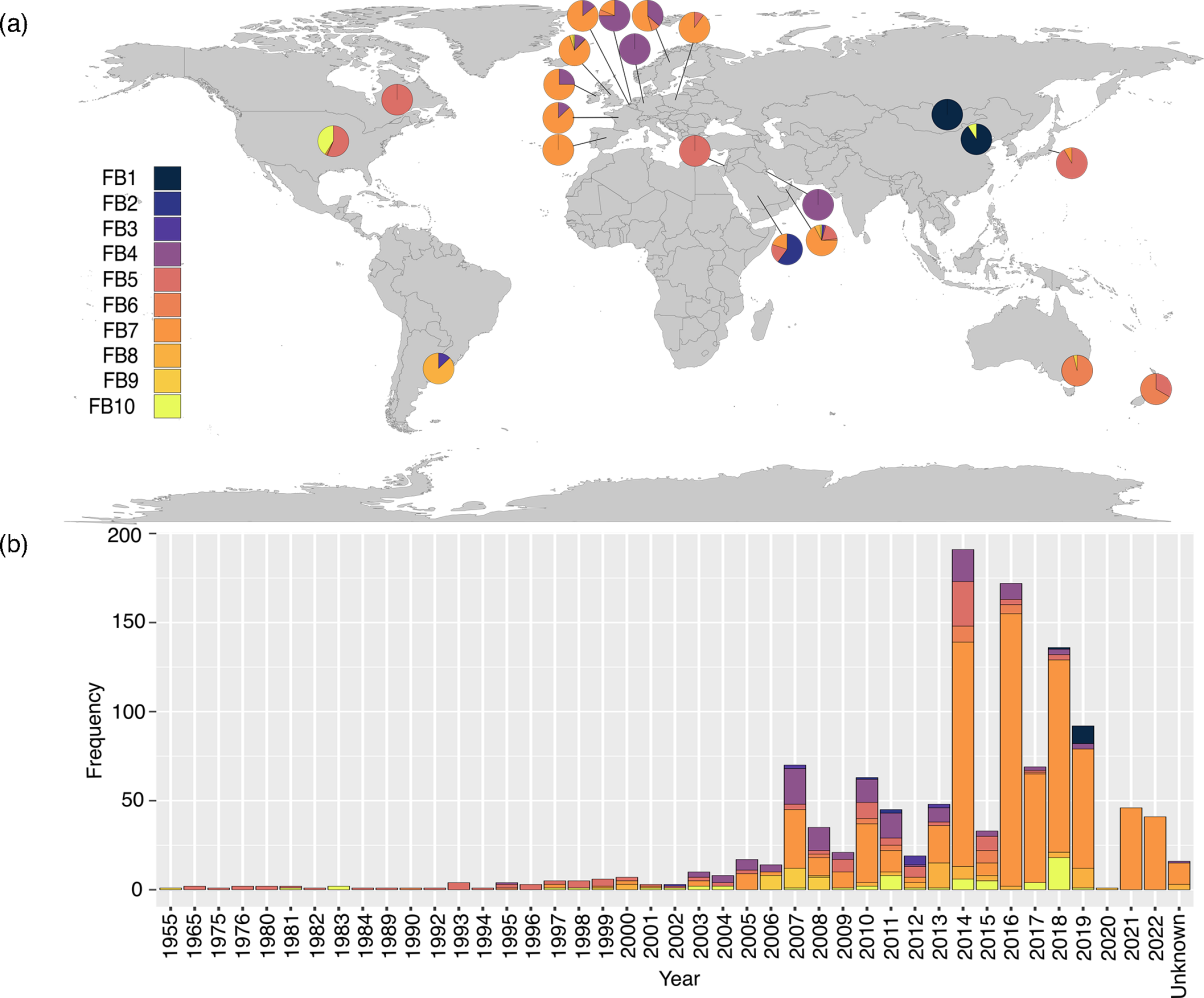
The global and temporal distribution of fastbaps clusters and STs. (a) Global fastbaps cluster distribution. Each pie chart is divided according to clusters present in the country associated with it. Clusters are numbered according to the left-hand legend. (b)Temporal distribution of fastbaps clusters identified between 1955 and 2022. Clusters are coloured to match the legend in (a).

Within the new fastbaps clusters, we found some evidence of geographical clustering (Fig. 1a, b). FB2 was composed of three identical isolates from Saudi Arabia that had previously clustered within BAPS6 [11]. Isolates from Argentina comprised 70% of FB3 (previously BAPS4). Within FB6, 26/31 (84%) of the isolates originated from Australia or New Zealand. Eighty-five per cent (47/55) of the isolates in FB8 (previously BAPS4) were isolated from horses in Argentina, and in FB9 (previously BAPS4), 27/28 (96%) isolates were collected from Lincolnshire in the UK during an outbreak. In FB10, 46/59 (78%) isolates were collected from the USA, although the majority of these were from a large outbreak in TX (Fig. 1a). We identified an increase in the FB7 cluster over time beginning in the early 2000s and continuing throughout the collection.

**Fig. 2.**
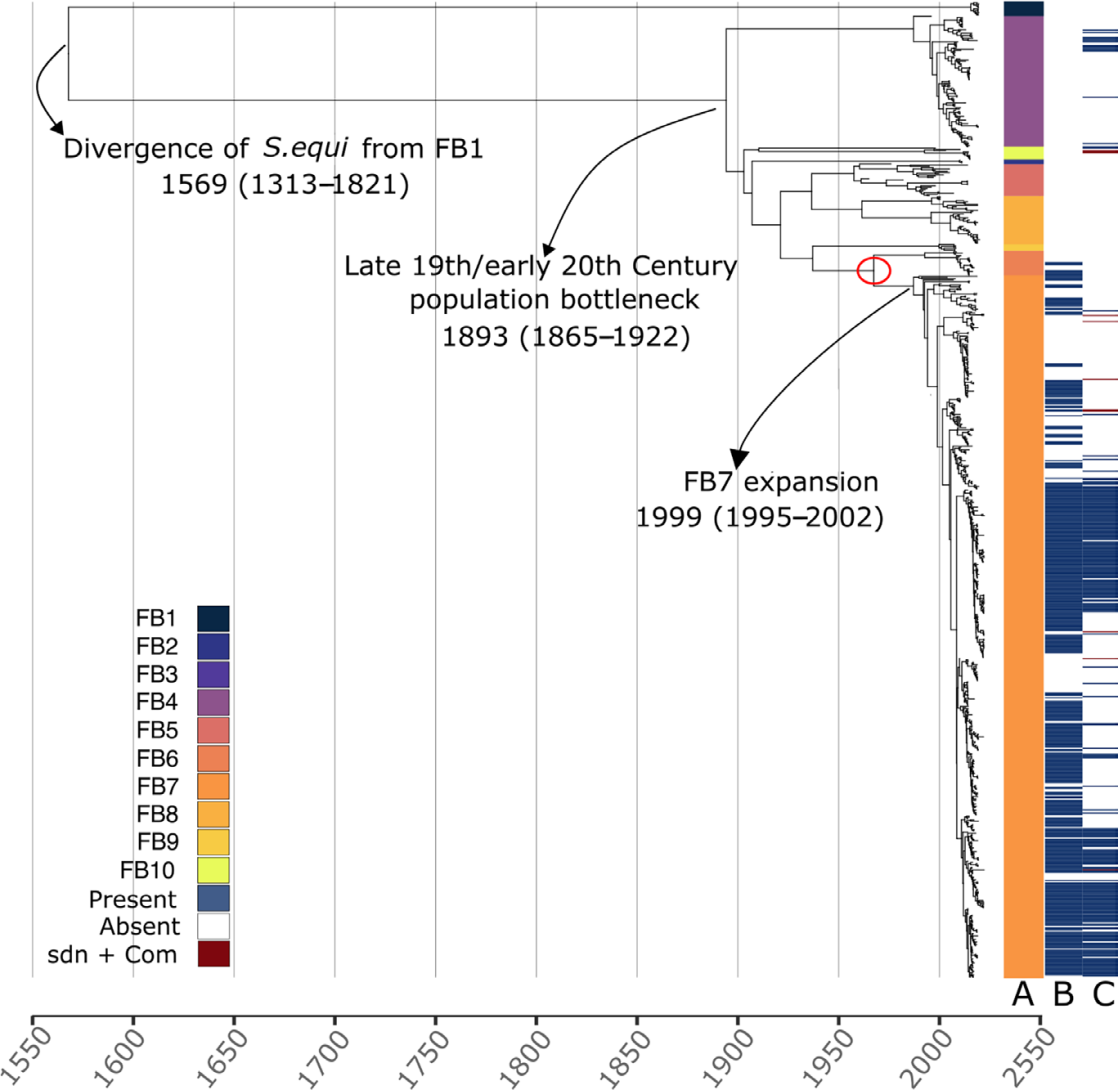
Bayesian phylogeny of global *S. equi.* MCC phylogeny demonstrates key evolutionary time points. Three evolutionary time points are highlighted with the estimated TMRCA stated and 95% Highest posterior density (HPD) for each in brackets. The red circle indicates a proposed gene gain event associated with the new prophage. Column A: fastbaps clusters, coloured according to left-hand legend and to match Fig. 1. Column B: the presence (blue) and absence (white) of the new prophage. The prophage was considered present if 90% or more of the genes were identified. Column C: the presence or absence of the *Sdn* gene. The presence of the gene is shown in blue and the presence with the addition of competence genes in red.

Of particular interest were the FB1 and FB7 clusters (Fig. 2). Eleven isolates were located on a deeply divergent branch in the phylogeny, which formed FB1 and were recovered from various locations within China. These isolates were collected during 2018 and 2019; however, their deeply branching position within the phylogeny shows that they have a much older last common ancestor with the rest of the collection, which all shared a last common ancestor around a century ago (Fig. 2). Of note is that these isolates were all collected from donkeys rather than horses. The second cluster of interest, FB7, represents a recent large expansion of a low-diversity lineage, particularly in the UK (Figs1b  3). These clusters are discussed in more detail below.

**Fig. 3.**
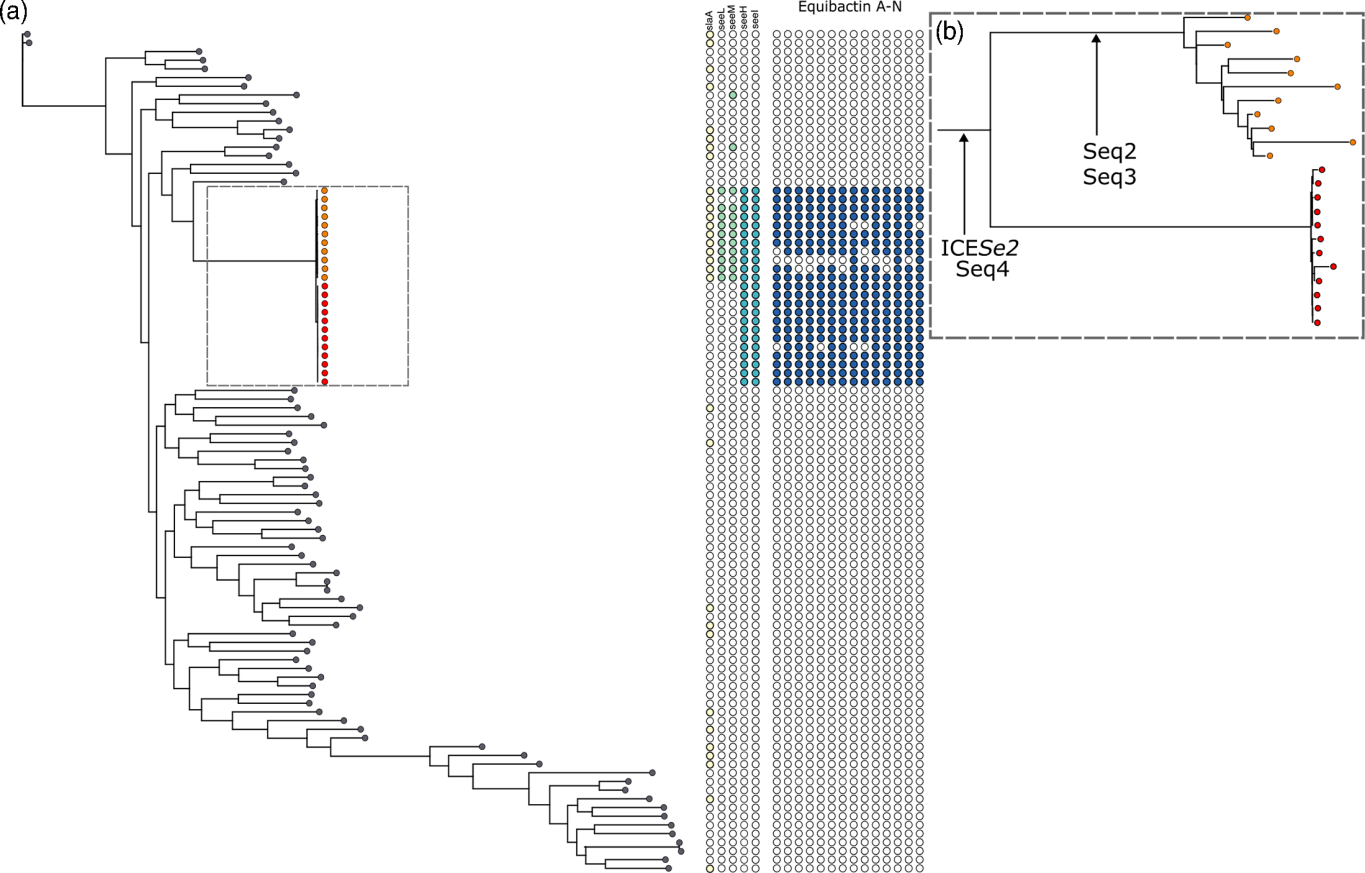
Acquisition of key mobile genetic elements in *S. equi.* (a) Core gene phylogeny of representative *S. zooepidemicus* and *S. equi* isolates. Grey terminal nodes indicate *S. zooepidemicus* isolates. Orange tips show the horse-centric *S. equi* population. Red tips show the donkey-centric FB1 isolates. Heatmap demonstrates the presence and absence of the prophage and ICE element marker genes within the collection. Filled, coloured circles show presence, and open circles absence, of the elements. Yellow circles indicate presence of *slaA,* the marker for ΦSeq2, light green circles indicate the presence of *seeL* and *seeM,* which are the markers for ΦSeq3, and blue circles show the presence of *seeH* and *seeI,* the markers for ΦSeq4. Dark blue circles show the presence of genes comprising the equibactin locus on the ICE*Se2* element. (**b)** Grey dashed box: focus on the *S. equi* isolates only from (a). The tips are coloured as for (a). Arrows indicate on which branch each MGE was acquired during the evolution of *S. equi* from an ancestral strain of *S. zooepidemicus.*

### Dating the phylogeny

By adding the FB1 isolates to the collection, the TMRCA of different lineages and dates of key divergence events can be more accurately estimated using Bayesian phylogenetics.

Firstly, the root-to-tip distances of branches in a phylogeny built using polymorphic sites in the core genome were plotted using TempEst. This gave a poor correlation between the accumulation of substitutions and time (*R*^2^=0.115), and the date-tip permutation test showed that this was not statistically significant (*P*=0.279). To improve the correlation prior to using BEAST2, we removed 371 isolates that fell more than 2 sd outside the mean based on residual data from the root-to-tip plot likely due to the quality of their assembly. Forty-nine isolates contributing to the poor root-to-tip correlation were published by Morris *et al.* The isolates were collected from an outbreak of strangles in horses at Texas A and M University in 2018 that occurred 5 months after a vaccine challenge trial. The trial had used a stored isolate that had been recovered from a horse with strangles in TX during 2011 as the challenge strain, which subsequently caused an outbreak in 2018, making this group of strains effectively genetically younger than their chronological age. This affected the branch length/date correlation in this part of the tree. The remaining isolates excluded by this process consisted mainly of poor-quality, highly fragmented assemblies. The final dataset consisted of 830 isolates, including both acute and persistent isolates, which produced a root-to-tip correlation of *R*^2^=0.207 and a significant date-tip permutation test (*P*<0.05 from 1000 permutations). The dataset passed a date randomization test where ten replicates with random date-tip permutations were used to analyse the clock rate and ucldMean in BEAST2.

The horse-centric *S. equi* population was estimated to have diverged from FB1 around 1570 (95% highest posterior density (HPD) 1313–1821). Our data refined the TMRCA of the horse-centric *S. equi* population, from the estimate of 1909 (HPD: 1819–1946) by Harris *et al.* [10] to earlier 1893, and we were able to reduce the 95% HPD range from 127 to 57 years (between 1865 and 1922), which still falls within the original HPD range (Fig. 2).

### Events before the bottleneck

Previous analysis of *S. equi* genomes identified a genetic bottleneck, suggesting a complete population replacement in the early twentieth century prior to the expansion of contemporary *S. equi.* The identification of divergent isolates forming FB1 enables us to separate the genetic events associated with the initial emergence of *S. equi* from those associated with the population bottleneck.

To investigate the genetic factors contributing to the formation of *S. equi*, we first created a pan-genome consisting of 74 representative *S. zooepidemicus* isolates, 11 *S. equi* from FB1 and 11 *S. equi* from the wider dataset. Eleven isolates from the wider dataset were chosen as representatives of the wider collection. One isolate was selected from each fastbaps cluster with the exception of FB7 where three were selected to represent the large expansion. We chose to include only a subset of the major *S. equi* groups as we were primarily concerned with identifying events at the root of the major *S. equi* expansion. The resulting phylogeny and annotation data were used to perform ancestral state reconstruction to determine when the MGEs in the collection were gained. To avoid multiple instances of cross-matching between phage machinery genes with high similarity, we used the virulence genes contained within the MGEs as proxy markers for prophage and ICE presence.

The MGE gene content differed between FB1 and the wider *S. equi* phylogeny (Fig. 3a). *seeH* and *seeI* (located on ΦSeq4) and genes constituting the equibactin locus (located on ICE*Se2)* were present in the genomes of both FB1 and the main *S. equi* clade; these genes were therefore acquired by *S. equi* prior to the divergence of FB1 (Fig. 3b). However, FB1 lacked *slaA* (marker for ΦSeq2) and *seeL* and *seeM* (markers for ΦSeq3), which were only acquired by the horse-centric *S. equi* population after the divergence of FB1. Our analysis therefore demonstrates a progressive acquisition of the MGEs rather than a single evolutionary event. This suggests that ΦSeq4 and ICE*Se2* are likely to contribute most significantly to the success of *S. equi* as a cause of strangles characterized by abscessation of the lymph nodes of the head and neck of both horses and donkeys, whilst the acquisition of ΦSeq2 and ΦSeq3 contributed to the further adaptation of *S. equi* to the horse, potentially to the detriment of fitness of the resultant horse-centric lineages in donkeys. To date, there have been no recorded cases of infection of horses with the donkey-centric FB1 lineage of * S. equi*, suggesting that this lineage may have poor fitness in horses. A single isolate of *S. equi* was recovered from a horse in China (China15) which clustered with other equine isolates in FB10 rather than within the FB1 group. This suggests that FB1 is not the only lineage identified in this geographical area. The presence and absence of these marker genes within the whole collection can be seen in Fig. 5, which demonstrates fixing of the genes within the population.

### Investigating the expansion of FB7 in the UK

The initial expansion of FB7 was first seen in the phylogenetic data from Harris *et al.* [10] in which it was identified as an expansion of the multilocus ST ST151. ST151 was first identified in our collection in 2005 where it accounted for 24% (4/17) of isolates collected. ST151 was identified in only 5 of the 21 countries in the collection: Belgium (10/21 isolates, 47%), the Netherlands (1/15 isolates, 6.6%), Poland (*n*=9/10 isolates, 90%), United Arab Emirates (*n*=19/114 isolates 16.7%) and the UK (*n*=496/732 isolates, 67.8%). Since 2010, ST151 accounted for 80.5% of UK sequenced isolates but only 10.6% of non-UK isolates in the collection from the same period. Since 2018, ST179 accounted for only 6.6% of isolates found in the UK, whilst ST151 accounted for 91.8% of isolates (ST179=17, ST151=236/257 and ST not identified=4). ST151 within FB7 has eclipsed ST179 and become the majority variant in the UK.

We estimate that FB7 first emerged in the UK in 1999 (95% HPD 1995–2002) from where it has since continued to expand (Fig. 2).

We used several analyses to identify potential drivers of the expansion of FB7 and the increased fitness identified in sub-lineages within FB7.

**Fig. 4.**
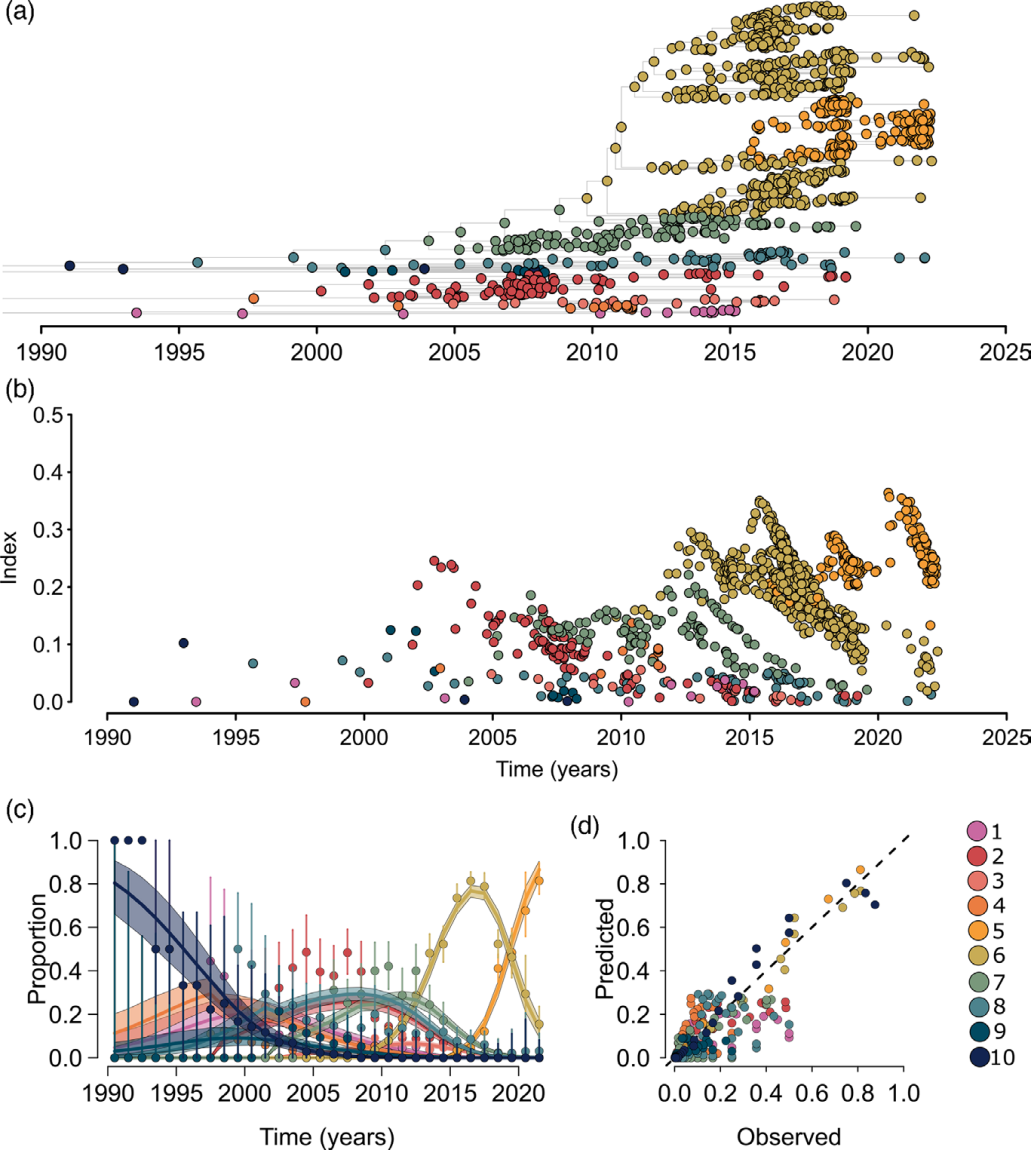
Diversity and fitness of *S. equi* isolates in the UK. (a) Bayesian dated phylogeny of *S. equi* isolates from the UK. Tips and nodes are coloured according to sub-lineages as detailed in the legend. (b)Fitness index of each isolate plotted over time. Each point refers to an isolate and is coloured according to the legend. (c)Fits to the proportion of each lineage through time. Dots represent data, and bars denote the multinominal 95% confidence intervals. (d)Predicted versus observed proportions, with predictions taken from the curve fitting in (c). The dotted line denotes the identity line. Colours across (a), (b), (c) and (d) represent the different lineages as shown in the right-hand legend.

We examined the fitness of this UK expansion in the context of other UK isolates since 2003. We have used an index that quantifies the fitness of each node in the tree [24]. This measure is based on the expectation that nodes sampled from an emerging fitter lineage will be phylogenetically closer than the rest of the population at that time. A higher index implies a lineage expansion in the population and therefore higher fitness. This approach was applied to the UK data subset to identify the set of discrete lineages circulating and quantify their fitness within the population. Within the UK population of *S. equi*, which contained FB clusters 4, 7, 8, 9 and 10, ten different sub-lineages were defined, based on their relative fitness index and genetic diversity (Fig. 4). Sub-lineages 1, 2, 3, 4 and 9 were not found in FB7 and were distributed throughout the remaining FB clusters. Within FB7, there were five sub-lineages (5, 6, 7, 8 and 10), indicating that multiple successive waves of sub-lineage expansions affected horses around the UK over the period studied.

Sub-lineage 5 was predicted to be the fittest within the dataset and has undergone the most recent expansion. However, the metadata associated with these strains did not reveal a particular reason for the increased success of this sub-lineage over others in FB7. These isolates originated from varied sources including guttural pouch samples and nasopharyngeal swabs. The isolates were also collected from across the UK between 2016 and 2022.

Ancestral state reconstruction identified the acquisition of a group of ~55 genes that consisted of multiple phage-related elements that may be influencing the FB7 expansion. The acquisition first occurred at the node ancestral to the expansion of FB7, and the group of genes was not identified in the collection before this point (Fig. 2, red circle). Of the 760 isolates within FB7, 531 (70%) of them were found to be carrying 90% or more of the acquired genes. Resolving the positioning of these genes proved problematic with the short-read data. At the assembly stage, large numbers of the genes were frequently assembled within ΦSeq2, giving the false impression that the structure of ΦSeq2 had changed. We were able to access long-read sequence data for 11 isolates, 4 of which were ST151, falling within the FB7 cluster and which had 90% or more of the genes identified via blast. Combined with our ancestral reconstruction and blast comparison data, we were able to identify a potential acquisition of a fifth prophage within *S. equi*, which was ~43 kbp in length and consisted of 52 coding sequences (Fig. S1A). A toxin-like gene was identified within this prophage, which shared the highest aa identity with a streptococcal DNA/RNA non-specific endonuclease. The endonuclease was most closely related to genes identified in *S. pyogenes* including the hypervirulent strain 1838, which caused toxic shock syndrome in a human [32], and strains NCTC8304 and NCTC8195.

A second potential driver for this widespread expansion in the UK may be the acquisition of the DNAase streptodornase (*sdn*), which was found in 321 (26.7%) isolates within the dataset in 2 differing genetic contexts. Firstly, 45 isolates contained the *sdn* gene with 4 competence genes situated downstream. With the exception of one cluster, these genes were found sporadically throughout the phylogeny though predominantly in early branching *S. equi* seen closer to the population sweep identified by Harris *et al.*

Secondly, the majority of isolates containing *sdn* (276/321, 86%) also encoded a paratox gene, and this *sdn* differed from the minority *sdn* associated with the competence genes by 1 synonymous SNP (Fig. S1B). This allele of *sdn* in this context was identified in three fastbaps clusters, FB4, 7 and 10, none of which cluster together within the phylogeny, indicating multiple acquisition events of this gene. The majority of these isolates (93%) were found within FB7, distributed among lineage fitness groups 5, 6, 7, 8 and 10. Sixty-five per cent of isolates carrying this gene were identified in lineage fitness group 5 (*n*=96), which was identified as the fittest group of the UK isolates, and lineage group 6 (*n*=130).

### MGE prevalence in the *S. equi* population

**Fig. 5.**
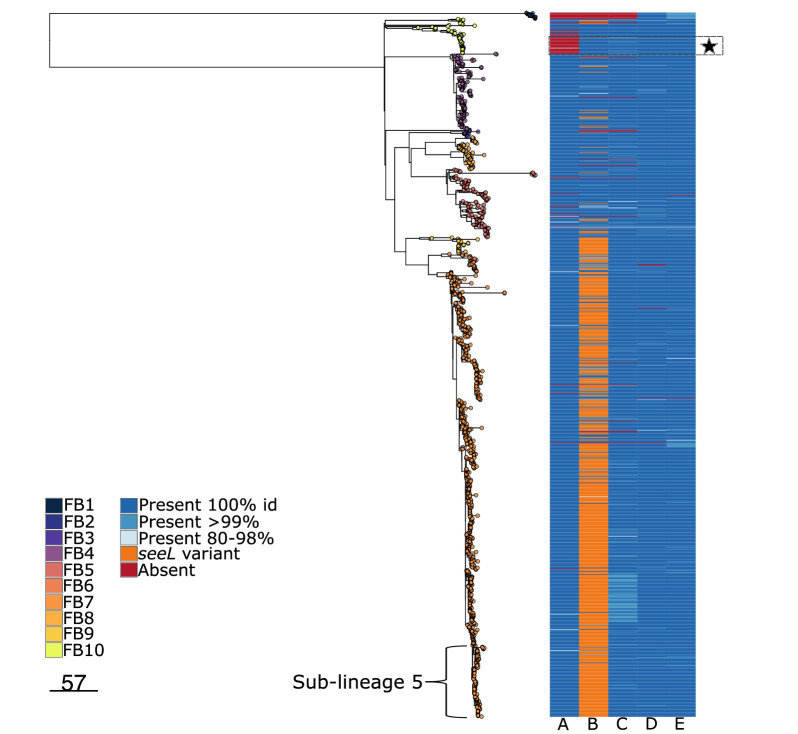
Core genome phylogeny of *S. equi*. Phylogenetic tree of the *S. equi* isolate collection. Tips are coloured according to fastbaps clusters and match those in previous figures. The right-hand heatmap demonstrates the presence and absence of prophage markers. Column A, *slaA*; column B, *seeL*; column C, *seeM*; column D, *seeH*; column E, *seeI*. The presence and absence are coloured according to the legend. The black star indicates the CVM outbreak cluster lacking slaA. Sub-lineage 5 is highlighted and corresponds to sub-lineage 5 in Fig. 4.

Following the acquisition of MGEs by *S. equi* during its evolution from *S. zooepidemicus,* the presence of these genes appears relatively stable across the dataset with a small number of exceptions (Fig. 5). *slaA* (the proxy marker for ΦSeq2) was absent (or present at less than 80% identity) in 57/1201 (4.7%) of isolates. The main contributors to this absence were 2 specific clusters within the population: 11 isolates in FB1 and 35 of the 59 isolates (59%) in FB10 (Fig. 5, black star). Isolates in this FB10 subcluster were first identified in the early 2000s in the Eastern and North-eastern regions of the USA (WA, ME and KY) where they were isolated from single horses until the same strain was identified as the cause of an outbreak in TX in 2011 (*n*=8). An isolate from this outbreak was then stored and utilized as an infection strain in a vaccine challenge at the College of Veterinary Medicine and Biomedical Sciences, Texas A and M University (CVM), during 2017 and 2018 [12]. Five months after the conclusion of the vaccine trial, an outbreak occurred in a different horse group at CVM, following co-mingling of a horse that had recovered from the experimental challenge. The authors concluded that a persistent infection of *S. equi* in a horse from the first phase of the vaccine trial was responsible for the outbreak. The isolates in FB10 were found to encode ΦSeq2; however, the *slaA* gene and a small number of adjacent phage genes had been lost from the MGE. In contrast, in FB1, there was no evidence of acquisition of ΦSeq2. These data further support that the equibactin loci, *slaA*, *seeH*, *seeI*, *seeL* and *seeM*, are required for the widespread success of *S. equi* in horses.

*seeH* and *seeI* were identified in 1196 (99.6%) and 1199 (99.8%) of isolates, respectively. The markers *seeL* and *seeM*, used to demonstrate the presence of ΦSeq3, were both present in 1174 (97.8%) of isolates. Interestingly, two nonsynonymous SNPs at adjacent aa positions have arisen in *seeL* and are found in most of the population, T668A and A672C, resulting in I223R and R224S, respectively (Fig. 5, column B). We infer that A672C occurred after T668A. Seven hundred thirty-three of one thousand two hundred one (61%) isolates in the collection encode these 2 mutations, occurring in all but 2 of the fastbaps clusters but predominantly in FB7 (661/733, 90%).

## Discussion

*S. equi* has evolved from the host generalist *S. zooepidemicus* to become a highly contagious host-specific equine pathogen with the ability to rapidly transmit globally, causing multiple outbreaks and considerable economic losses. We have combined multiple existing datasets with new genomes to create the largest dataset for analysis to date and shed unprecedented light on the evolutionary pathway of *S. equi.*

The use of MLST in *S. equi* analyses does not provide the resolution required to make epidemiological decisions about the pathogen. To circumvent this issue, we utilized fastbaps to examine our larger dataset. fastbaps produced 10 clusters across the 1201 isolates, and the addition of new isolates to this dataset highlighted 2 particular clusters, FB1 and FB7.

FB1 was highly divergent compared to the rest of the *S. equi* collection. Interestingly, these isolates were collected from donkeys primarily in China in 2018 and 2019. The phylogenetic positioning of these isolates provided an opportunity for us to investigate events that occurred before the early twentieth-century population bottleneck [10]. The presence of four prophages and two ICEs unique to *S. equi* suggests that the acquisition of these MGEs was crucial to the evolution of *S. equi.* By using ancestral state reconstruction, we have shown that the arrival of these MGEs was sequential rather than a single event. ΦSeq4 and ICE*Se2* were acquired by *S. equi* prior to the separation of FB1 from FB2–10, i.e. before the population replacement in the early twentieth century. The acquisition of these two MGEs enabled *S. equi* to begin to expand as a pathogen of hosts within the *Equidae* family, which includes donkeys as well as horses. This observation is supported by earlier work examining the functionality of the superantigens encoded by these MGEs.

The superantigens, SeeH and SeeI*,* encoded by genes on ΦSeq4, share 99% aa sequence similarity with the *S. pyogenes* genes SpeH and SpeI. Paillot *et al.* [33] previously demonstrated the contribution to mitogenicity and host immune response for each superantigen to better understand their role in pathogenicity. SeeI, SeeL and SeeM all produced a dose-dependent response in equine CD4 T lymphocytes and gamma interferon (IFN-*γ*), with SeeM demonstrating the most potent activity. Interestingly, SeeH did not stimulate the same immune response in the PBMC from ponies or other equine breeds. It did however induce *in vivo* proliferation of PBMC from donkeys. This was found to be due to the absence of equine cells carrying the appropriate T-cell receptor V*β* chains and/or MHC class II molecules that were recognizable by SeeH. Due to the functional redundancy provided by the three other superantigens, SeeH may not have encountered the selective pressure to adapt to equine PBMCs. Our data demonstrate that FB1, which was found solely in donkeys, carried *seeI* and *seeH* genes as cargo on ΦSeq4, but not *seeL* or *seeM* genes. The lineage was therefore able to successfully infect donkeys due to the influence of the SeeH superantigen, whereas the effect of this protein in horses becomes redundant in the face of lineages producing the more potent SeeL and SeeM. It would be enlightening to collect further samples from donkeys with strangles-like disease to examine this behaviour on a larger scale. The SeeL N^223^, S^224^ variant was encoded by some isolates across eight of the ten fastbaps clusters, including 90% of those in the fitter FB7 cluster, raising the possibility that these mutations also contribute to the adaptation of *S. equi* to horses.

ICE*Se2* contains the equibactin locus, a group of genes likely to be responsible for improving iron acquisition in *S. equi*, which has overall similarity to the genes involved in synthesizing the iron scavenging yersiniabactin system seen in *Yersinia pestis* [9]. Mutations in yersiniabactin biosynthesis genes result in strains unable to cause bubonic plague whilst equibactin production was required for the full virulence of *S. equi* in ponies and the absence of equibactin production was associated with long-term carriage [10]. Whilst *S. zooepidemicus* is adept at causing respiratory infections in horses and can be identified in ~25% of apparently healthy sport horses [34,35], reports of severe abscessation in equines are less common than in infections with *S. equi.* Therefore, it seems that *S. equi* is reliant on this iron-acquisition system to establish deep-seated infections that can then be transmitted to naïve animals. Our data demonstrate that the acquisition of this system was part of the early events in the evolution of *S. equi* from *S. zooepidemicus*, suggesting that this is a fundamental part of the * S. equi* lifestyle. However, the ancestor represented by FB1 has clearly been replaced in most of the global horse population, indicating that the production of all four of the superantigens as well as the equibactin locus may provide the later-evolving lineage with a greater advantage for widespread transmission and success in populations of horses.

Secure adhesion of *S. equi* particularly to the ciliated pseudostratified columnar epithelium of the nasopharyngeal tonsils is key to prevent mucociliary clearance and/or clearance by the innate immune defences of the tonsil [36]. *slaA*, found on ΦSeq2, has 98% similarity to the phospholipase A_2_ gene found in *S. pyogenes.* This virulence factor is thought to be key to streptococcal colonization of the upper respiratory tract. Our data show that *slaA* was absent from FB1 isolates and 59% of FB10 isolates, suggesting that its acquisition was important for the global replacement of these early lineages and the success of the later lineages. The prevalence of FB1 in donkeys outside of this work however is unknown due to the lack of screening. The FB10 lineage had been seen in sporadic, single cases since the early 2000s but had not shown widespread transmission. The *slaA-*absent FB10 sub-lineage then caused one outbreak in 2011 in a ranch environment and a second outbreak in a naïve herd of horses at the College of Veterinary Medicine and Biomedical Sciences, Texas A and M University (CVM), in 2018 [12]. The horses were exposed to a horse previously enrolled in a vaccine trial using the 2011 ranch strain as the challenge strain. Despite being clinically negative for *S. equi*, this horse appears to have seeded an outbreak, although naïve horses did not develop disease until 5 months after the introduction of the trial horse. The authors concluded that persistent carriage from the first phase of the vaccine trial was responsible for the outbreak. An alternative explanation is that the lack of *slaA* in the vaccine challenge strain slowed the progression of the outbreak, resulting in the 5-month time lag. The absence of the *slaA* gene may have impacted the pathogens’ ability to attach and infiltrate tissues key to the establishment of infection.

The acquisition of ΦSeq2 and ΦSeq3 in the population correlates with the replacement of the early lineages with those responsible for *S. equi*’s global expansion since the early twentieth century. Overall, these data suggest that the acquisition of ΦSeq4 and ICE*Se2* was important for the early evolution of *S. equi* from *S. zooepidemicus*, allowing it to establish as a pathogen of equines. However, the subsequent acquisition of ΦSeq2 and ΦSeq3 by a specific lineage allowed the expansion and replacement of earlier lineages.

We utilized the BEAST2 [23] package to determine when this division of lineages may have occurred. The removal of isolates from Morris *et al.* [12] was required as they confounded the root-to-tip correlation analysis. This is likely due to the 2018 CVM outbreak being seeded by a vaccine trial isolate collected in 2011. Once these data had been removed, along with isolates with more fragmented assemblies, a significant correlation between root-to-tip distance and date of isolation was observed. Analysis of the dated Bayesian data using the MCC phylogenetic tree indicated that FB2-10 diverged from FB1 in 1569 (95% HPD 1313–1821). This date estimation is after the earliest recorded mentions of strangles-like disease. Strangles was first described by Jordanus Rufus, a knight of Emperor Fredrick II of Hohenstaufen, King of Naples and Sicily, around 1251 [1], suggesting that this may have been caused by very early lineages of *S. equi*. The disease is then also reported in detail in the first edition of ‘*Le parfait marechal*’ (The Perfect Stable Master), which was written in 1664 by Solleysel [37]. This precedes the population sweep described below and may therefore have been caused by an intermediary lineage from FB1 that led to the contemporary strains of *S. equi*.

We refined the original estimate of 1909 (95% HPD 1819–1946) for the population sweep first identified by Harris *et al.* [10]. A TMRCA estimate of 1893 (95% HPD 1865–1922) was identified and has reduced the 95% HPD by 70 years. Harris *et al.* [10] hypothesized that the population sweep driven by this lineage occurred during a time when horses were heavily involved in global conflicts and particularly in World War I. At the peak of this conflict, 1000 horses per day were moved between the UK and the USA, creating numerous opportunities for the transmission of fitter *S. equi* lineages to and between naïve horses [38]. Our ancestral dates remain consistent with this driver, but we should also consider conflicts from the fourth quarter of the eighteenth century as catalysts for * S. equi* success; for example, conflicts involving multiple countries and territories include both the first (1880–1881) and second Boer war (1899–1902) and the Boxer Rebellion (1899–1901).

ST151, which differs from ST179 by one allele, has been steadily expanding in prevalence since its first identification in the UK [5]. Continued expansion of this ST has been identified, where it has increased in prevalence from 16.8% [8] to 23.3% [11] in global data. Our data, which contain the largest collection of *S. equi* to date, show that ST151 now accounts for 44.5% (535/1201) of isolates globally, although 92.7% of these isolates originate from the UK. Almost all ST151 isolates fell within FB7. Isolates from 12 countries were found within FB7; however, isolates from the UK comprised 76.7% of the isolates in this cluster. Our Bayesian analysis estimated that the FB7 ancestor emerged in 1999 and the lineage began to expand from this point onwards. We sought to identify potential reasons for this successful expansion.

Ancestral state reconstruction and long-read sequence data enabled us to identify the potential gain of a new prophage, which has been retained in a large proportion of FB7 *S. equi* isolates. Streptococcal species contain a myriad of prophages that impact upon their pathogenicity. Acquisition of these entities and their cargo are responsible for epidemiological changes in multiple streptococci. *S. pyogenes* (Group A *Streptococcus*) M1 serotype rapidly spread to become the dominant serotype following three horizontal gene transfer events. The lineage gained phages carrying the superantigen SpeA and DNAase *sdaD2* and a 36 kb region that caused the upregulation of two virulence genes, *slo* and *nga*, which coincided with its emergence as an epidemic lineage [39]. More recently, three genomic islands have been identified in *Streptococcus suis*, which are thought to be influential in the spread of pathogenic lineages [40]. Indeed, the gain of ΦSeq2 and ΦSeq3 appears to be involved in the successful global expansion of modern *S. equi* in horses but not donkeys. The emergence of FB7, associated with the acquisition of an additional phage-borne virulence factor, may be the beginning of a new population sweep by this lineage.

There are some caveats to consider in this work. The project was interrupted by the closure of the Animal Health Trust, which occurred at the same time as the global severe acute respiratory syndrome coronavirus 2 pandemic. As a result of these disruptions, the dataset does not contain isolates from countries other than the UK after 2020. This may impact our observations of the spread of FB7. However, the lineage had already spread to multiple countries, and considering the transmissibility of *S. equi*, the FB7 lineage is likely to now be much more widespread. SES has provided the first in-depth, contemporary set of data from the UK; however, it also generates a geographical bias in the dataset, which may affect our conclusions.

Here, we have combined multiple sources of published and novel genome sequence data to create the largest collection of *S. equi* genomes to date. We have used these data to build on previous understanding and address some of the emerging knowledge gaps. We have identified an early-branching lineage of *S. equi* that emerged before the bottleneck in the early twentieth century and used this to identify genetic changes associated with the initial emergence from *S. zooepidemicus* and those associated with the selective sweep that led to this bottleneck. We have also identified a recently emerged lineage that is expanding in the *S. equi* population and describe novel genes and variants associated with this expansion. These results are of clinical importance and will benefit future surveillance and vaccine development efforts.

## Supplementary material

10.1099/mgen.0.001366 Uncited Supplementary Material 1.

10.1099/mgen.0.001366 Uncited Table S1.

## Abbreviations

BAPS: Bayesian Analysis of Population Structures
HPD: highest posterior density
ICE: integrative conjugative elements
MCC: maximum clade credibility
MGEs: mobile genetic elements
MLST: multilocus sequence typing
SES: surveillance of equine strangles
ST: sequence type
TMRCA: time to most recent common ancestor
