# Corrigendum: Progressive evolution of Streptococcus equi from Streptococcus equi subsp. zooepidemicus and adaption to equine hosts

**DOI:** 10.1099/mgen.0.001426

**Published:** 2025-06-18

**Authors:** Hayley J. Wilson, Jianbao Dong, Andries J. van Tonder, Christopher Ruis, Noémie Lefrancq, Abigail McGlennon, Carla Bustos, Sara Frosth, Albertine Léon, Adam M. Blanchard, Matthew Holden, Andrew S. Waller, Julian Parkhill

**Affiliations:** 1 PHG Foundation, linked exempt charity of University of Cambridge, Cambridge, UK; 2 Department of Veterinary Medicine, University of Cambridge, Cambridge, UK; 3 College of Animal Science and Technology, Qingdao Agricultural University, Qingdao, PR China; 4 Victor Phillip Dahdaleh Heart & Lung Research Institute, University of Cambridge, Cambridge, UK; 5 Department of Genetics, University of Cambridge, Cambridge, UK; 6 Royal Veterinary College, Hatfield, Hertfordshire AL9 7TA, UK; 7 EIDS, Department of Veterinary Medicine, University of Cambridge, Cambridge, UK; 8 Facultad de Ciencias Veterinarias, Cátedra de Enfermedades Infecciosas, Universidad de Buenos Aires, Buenos Aires, Argentina; 9 Consejo Nacional de Investigaciones Científicas y Técnicas (CONICET), Buenos Aires, Argentina; 10 Department of Animal Biosciences, Swedish University of Agricultural Sciences, P.O. Box 7023, 750 07 Uppsala, Sweden; 11 LABÉO, Research Department, St Contest, Caen, France; 12 Normandie Univ, UNICAEN, INSERM, DYNAMICURE UMR 1311, Caen, France; 13 School of Veterinary Medicine and Science, University of Nottingham, Sutton Bonington, UK; 14 Infection Group, School of Medicine, University of St Andrews, North Haugh, St Andrews, UK; 15 Intervacc AB, Stockholm, Sweden

In the published version of this article, the name of Jianbao Dong was spelled incorrectly. In the previous version, the name appeared as Jiangbao Dong and has now been corrected to Jianbao Dong in the original version.

The authors apologise for any inconvenience caused.

