# Supplementary material for: Progressive evolution of Streptococcus equi from Streptococcus equi subsp. zooepidemicus and adaption to equine hosts
Source: Microb Genom. 2025 Mar 28;11(3):001366. doi: 10.1099/mgen.0.001366 (PMC12453393; doi:10.1099/mgen.0.001366)
Supplement: Uncited Supplementary Material 1. [file mgen-11-01366-s001.pdf]

Materials and Methods:

Bacterial isolates: Two hundred and twenty-four of the 639 isolates from Mitchell et al were from the original dataset in Harris *et al.*[10] . Seventeen additional genome sequences of isolates collected from horses in France between 2016 and 2019 were provided by Dr Albertine Léon. The genomes of 41 *S. equi* isolates recovered from horses in Argentina between 2010-2020 were submitted by Dr Carla Bustos. The genomes of 12 isolates recovered from donkeys and a horse in China between 2018 and 2019 were submitted by Jiangbao Dong. A collection of 443 genomes from isolates recovered from horses in the UK collected as part of the SES network during 2016 to 2022 were also incorporated into the collection. Finally, the genome sequences of 49 isolates that were recovered from horses in Texas or Kentucky between 2011 and 2018 that were used in the dataset from Morris *et al*.[12], 2020, were also included.

Sequence read quality control: Briefly, the pipeline uses FastQC (https://fastbaps.bioinformatics.babraham.ac.uk/projects/fastqc/) to read the fastq files for each isolate and Fastq-scan (https://github.com/rpetit3/fastq-scan) is then used to calculate summary statistics. The pipeline then uses Fastp[18] to trim reads for quality and adapter sequence. Taxonomic labels are assigned to the reads by Kraken 2[19] followed by refinement through re-estimation of taxonomic abundance of samples by Bracken[19]. KrakenTools[19] was optionally used at this stage to extract isolates based on chosen parameters. Finally, MultiQC[20] was used to present quality data for each isolate.

Genomic comparisons between *S. zooepidemicus* and *S. equi*

The *S. zooepidemicus* and *S. equi* isolates were annotated using Bakta[23] with default settings. Panaroo[24] was used to create a core gene alignment of all the *S. zooepidemicus* isolates. Polymorphic sites were identified using snp-sites[25] and a phylogenetic tree was created using IQTree[20] with the settings described above. fastbaps[18] analysis produced 64 clusters of which a representative for each cluster was selected based on assembly quality. If clusters contained more than 80 isolates (i.e., more than 10% of the total dataset) then one representative per subcluster was taken forward. This was the case in one large cluster. Multiple fastbaps assignments comprised single isolates only. The final number of isolates taken forward to compare with *S. equi* was 74. One isolate per fastbaps cluster for *S. equi* and all FB1 isolates were also included in this analysis (Supplementary Data 1). The annotations of the selected isolates were then used as input for Panaroo[24] to create a core gene alignment of both species. Polymorphic sites were identified using snp-sites[25] and a phylogenetic tree created using IQTree[20] with the same settings described above.

Dating the phylogeny: Firstly, we checked the linear regression of root-to-tip distance versus isolation date using TempEst[31]. The main dataset produced a poor correlation (described in the results); therefore 371 isolates falling more than two standard deviations outside of the mean in the TempEst[31] residual plot were removed, leaving a new dataset of 830 isolates . A multifasta alignment of polymorphic sites was created using the same methodology as for fastbaps clustering**.** This alignment was used to create the xml input files for BEAST2[30] using BEAUTI[32]. We employed a Hasegawa-Kishono-Yano (HKY) substitution model, a relaxed log normal clock model with a lognormal prior on the clock rate with mean 5.22645×10^{-7} (based on the slope rate in the TempEst[31] data) and standard deviation 0.5. We used a coalescent Bayesian skyline tree prior.

Fitness Index: The framework enables evaluation of recent population dynamics in addition to the information gathered about long-term evolution identified by BEAST. The framework enables automatic detection of lineages based on inferred fitness and evolutionary relationships. It allows quantification of the relative fitness advantage of new lineages which in turn aids in the identification of potential drivers of this emergence. Specific parameters that were used for *S. equi* were: mutation rate = $4.13\text{x}10^{-7}$, genome length = $1.9\text{x}10^{6}$, timescale = 1, t_wind = 1 year, and minimum number of

isolates per group = 5. Lineage fitness quantification was performed by fitting the proportion of each lineage through time using a multinomial logistic model that considers the birth of lineages. We assume each lineage has a constant fitness through time, defined as its relative growth rate in the population. The model was implemented in Stan, using the cmdstanr package[34].

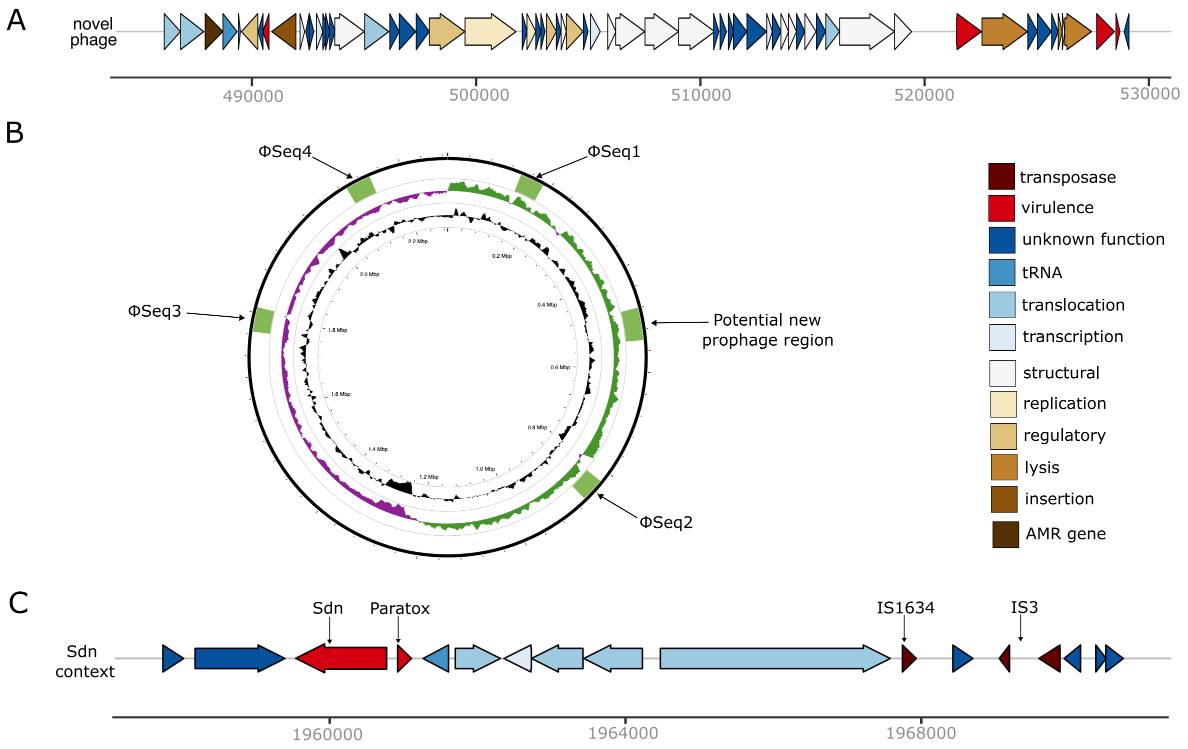

Figure S1: Visualisation of potential drivers of FB7 expansion. A: Gene schematic of the potential new prophage acquired in FB7. Genes are coloured according to annotated functions as detailed in the right-hand legend. Scale is in base pairs. **B:** Circular diagram of *S. equi* genome S034 produced using PHASTEST (https://phastest.ca/) demonstrating the addition of a fifth potential new prophage in the context of known prophages ΦSeq1-4. Black inner ring shows GC content. Green and purple ring shows GC skew. **C:** Gene schematic of *Sdn* and the genes surrounding it. Genes are coloured according to annotated functions as detailed in the right-hand legend. Scale is in base pairs.
